# Donor T-Cell Repertoire Profiling in Recipient Lymphoid and Parenchyma Organs Reveals GVHD Pathogenesis at Clonal Levels After Bone Marrow Transplantation in Mice

**DOI:** 10.3389/fimmu.2021.778996

**Published:** 2021-12-07

**Authors:** Yongxia Wu, Jianing Fu, Haizhen Wang, Xue-Zhong Yu

**Affiliations:** ^1^ Department of Microbiology and Immunology, Medical University of South Carolina, Charleston, SC, United States; ^2^ Columbia Center for Translational Immunology, Department of Medicine, Columbia University, New York, NY, United States; ^3^ Department of Cell and Molecular Pharmacology and Experimental Therapeutics, Medical University of South Carolina, Charleston, SC, United States; ^4^ Hollings Cancer Center, Medical University of South Carolina, Charleston, SC, United States; ^5^ Department of Medicine, Medical University of South Carolina, Charleston, SC, United States

**Keywords:** bone marrow transplantation, allogeneic hematopoietic stem cell transplantation, TCR repertoire, graft-versus-host disease, T cells

## Abstract

The diversity and composition of T-cell receptor (TCR) repertoire, which is the result of V, (D), and J gene recombination in TCR gene locus, has been found to be implicated in T-cell responses in autoimmunity, cancer, and organ transplantation. The correlation of T-cell repertoire with the pathogenesis of graft-versus-host disease (GVHD) after allogeneic hematopoietic cell transplantation remains largely undefined. Here, by utilizing high-throughput sequencing of the genes encoding TCRβ-chain, we comprehensively analyzed the profile of T-cell repertoire in recipient lymphoid and GVHD target organs after bone marrow transplantation (BMT) in mice. In lymphoid organs, TCR diversity was narrowed, accompanied with reduced numbers of unique clones while increased accumulation of dominant clones in allogeneic T cells compared to syngeneic T cells. In an individual allogeneic recipient, donor-derived TCR clones were highly overlapped among tissue sites, and the degree of overlapping was increasing from day 7 to 14 after allogeneic BMT. The top clones in peripheral blood, gut, liver, and lungs were highly mutually shared in an allogenic recipient, indicating that blood has the potential to predict dominant clones in these GVHD target organs. T cells in GVHD target organs from allogeneic recipients had fewer overlapped clones with pre-transplant donor T cells compared to those from syngeneic recipients. Importantly, the top 10 clones in allogeneic recipients were not detectable in pre-transplant donor T cells, indicating clonal expansion of rare rearrangements. Interestingly, even starting from the same pool of donor repertoires, T cells had very few overlapped clones between each allogeneic recipient who developed completely different dominant clones. We were only able to trace a single clone shared by three replicate allogeneic recipients within the top 500 clones. Although dominant clones were different among allogeneic recipients, V26 genes were consistently used more frequently by TCR clones in allogeneic than syngeneic recipients. This is the first study to extensively examine the feature of T-cell repertoire in multiple lymphoid and parenchyma organs, which establishes the association between T-cell activation and GVHD pathogenesis at the level of TCR clones. Immune repertoire sequencing-based methods may represent a novel personalized strategy to guide diagnosis and therapy in GVHD.

## Introduction

Allogeneic hematopoietic cell transplantation (allo-HCT) is a viable option to cure hematological malignancies such as leukemia, myeloma, and lymphoma (1). The efficacy of allo-HCT relies on the ability of lymphocytes in the donor graft to eliminate remaining malignant cells following radiation and/or chemotherapy, termed the graft-versus-leukemia (GVL) effect. The success of allo-HCT is undermined by the development of graft-versus-host disease (GVHD), which occurs in 30%–70% of transplant recipients and remains a prominent cause of morbidity and mortality (2). GVHD is a tissue-destructive process involving damage in classic target organs, including skin, gut, liver, lungs, and the newly demonstrated central nervous system.

T cells are the driving force for inducing GVHD by secreting cytolytic molecules and inflammatory cytokines (3). T-cell antigen receptors (TCR) play key roles in recognition of antigen–major histocompatibility complex (MHC) complex during T-cell activation. The variable region of TCRα is encoded by variable (V) and joining (J) genes, while TCRβ is additionally encoded by diversity (D) genes. V(D)J recombination during T-cell development causing TCR variability enables the immune system to discriminate between self and non-self. Complementary determining region 3 (CDR3), encoded by the junctional region of V(D)J, is highly variable and the direct binding area with peptide antigen (4).

TCR repertoire had been found to affect T-cell response in a wide range of diseases or conditions, including cancer, autoimmune disease, solid organ transplantation, and HCT. Detection and tracking of donor-reactive T-cell clones provides a new mechanism of tolerance induction in solid organ transplant (5, 6). Reduced diversity of TCRβ repertoire in tumor infiltrate lymphocytes correlates with clinical response to anti-PD-1 therapy in patients with metastatic melanoma (7). In patients of allo-HCT, the recovery of TCRβ repertoire is made distinct by sources of donor graft in that cord blood-graft recipients approximated the TCR diversity of healthy individuals 6 months later, whereas the recipients of T-cell-depleted peripheral blood stem cell grafts had 28- to 14-fold lower T-cell diversities (8). Reduced TCR diversity was associated with both tumor relapse and GVHD development in patients following HCT (9). After BMT, repertoire evolved to a more donor-like state in recipients who did not develop GVHD as opposed to those who had GVHD who were abundantly reconstituted with pre-transplant low-frequency donor clones (10). In recipients of myeloablative, HLA-matched allogeneic BMT using PTCy for GVHD prevention, TCRβ clones contained herpesvirus- or alloantigen-specific CD8 T cells. Interestingly, posttransplant TCRβ and immunoglobulin heavy chain locus repertoires were unique to each patient (11). In addition, TCR repertoire diversity and overlapping in tissue sites were related to steroid response in GVHD patients (12). Therefore, profiling the TCR repertoire can be used to develop new diagnostic and prognostic markers in these diseases.

Using next-generation sequencing of TCRβ chain locus, we defined the feature of T-cell repertoires in mice after syngeneic or allogeneic bone marrow transplantation (BMT). We extensively studied post-transplant T-cell repertoire in multiple lymphoid and non-lymphoid GVHD target organs, including peripheral blood, spleen, peripheral lymph nodes (pLN), mesenteric lymph nodes (mLN), liver, lungs, gut, and skin. Our study provides a spectrum analysis of T-cell repertoire recovery across broad tissue sites after BMT and further develops its association with T-cell pathogenesis in GVHD induction.

## Materials and Methods

### Mice

C57BL/6 (B6) were purchased from the National Cancer Institute (NCI, Frederick, MD). Rag1^-/-^ B6 and Rag1^-/-^ BALB/c mice were purchased from Jackson Laboratory (Bar Harbor, ME). All mice were housed in a pathogen-free facility at the American Association for Laboratory Animal Care-accredited Animal Resource Center at Medical University of South Carolina (MUSC). All animal studies were carried out under protocols approved by the Institutional Animal Care and Use Committee at MUSC.

### BMT

Major histocompatibility complex (MHC) mismatched (B6→ Rag1^-/-^ BALB/c) and MHC matched (B6→ Rag1^-/-^ B6) murine BMT models were carried out. Briefly, recipient mice (8–10 weeks of age) were conditioned with total body irradiation (TBI) administered 550 cGy (Rag1^-/-^ BALB/c) or 750 cGy (Rag1^-/-^ B6) using an X-RAD 320 X-ray Irradiator (Precision X-ray Inc., North Branford, CT). Purified T cells together with bone marrow (BM) cells isolated from Rag1^-/-^ B6 were i.v. injected into these recipient mice within 24 h after TBI. On day 7 and day 14 after BMT, recipients were sacrificed, and tissues were harvested for gDNA isolation.

### T-Cell Purification

Donor T cells were purified from pooled spleen and lymph node in B6 mice by negative selection to remove non–T cells including B cells, natural killer (NK) cells, DCs, macrophages, and granulocytes. Briefly, non–T cells were indirectly magnetically labeled by using a cocktail of biotin-conjugated Abs against CD45R (B220) (eBioscience, clone RA3-B2), CD49b (DX5) (eBioscience, clone DX5), CD11b (Mac-1) (eBioscience, clone M1/70), and Ter-119 (eBioscience, clone Ter-119), as well as anti-biotin MicroBeads (Miltenyi Biotec). Isolation of T cells was achieved by depletion of the magnetically labeled cells.

### TCRβ Sequencing and Data Analysis

Sample DNA from recipient tissues was extracted using Qiagen DNeasy™ Blood & Tissue Kit as detailed by the manufacturer. A260/280 (1.8–2.0) and A260/230 (2.0–2.2) were measured ensuring good-quality DNA. The input gDNA for lymphoid and non-lymphoid organs were 960 and 3200 ng/replicate sample, respectively. A two-step PCR was performed using immunoSEQ mmTCRB kit (Adaptive Biotechnologies) to amplify the highly variable CDR3 region (step1) and add barcodes and adaptor sequences for sequencing (step 2). The generated library was sequenced *via* the Illumina MiSeq. Sequence data were later retrieved from Adaptive Biotechnologies’ ImmunoSEQ software. PCR amplification, read sequencing, and mapping, with bias correction and internal controls, were performed by Adaptive Biotechnologies, returning tabulated productive template counts corresponding to unique productive CDR3 sequences across all samples. Sequence data were analyzed using the combination of ImmunoSEQ software and R. Unless otherwise specified, unique sequence is defined by CDR3 nucleotide + V gene + J gene using Adaptive Biotechnologies’ “Combined Rearrangement analysis”.

### Statistical Analysis

GraphPad Prism was used to perform statistical analysis. Normality of data was assessed, and the statistical significance was determined with two-tailed unpaired Student’s *t*-test and two-way ANOVA (Sidak) to determine statistical significance (*p* < 0.05).

## Results

### TCR Diversity Was Narrowed and Top Clones Were Enriched in Allogeneic T Cells

To study the feature of TCR repertoire of donor-derived T cells in allogeneic and syngeneic recipients, we performed BMT by transferring the same donor T cells derived from C57BL/6 (B6) mice into irradiated Rag1^-/-^ BALB/c (allogeneic) and Rag1^-/-^ B6 (syngeneic) recipients. We used Rag1^-/-^ recipients and Rag1^-/-^ BM to accurately study T-cell clones that are donor-derived, mature T cells. Two time points, day 7 and day 14 after BMT, were selected for analyzing TCR repertoire in recipient peripheral blood, spleen, peripheral lymphoid nodes (pLN), mesenteric lymphoid nodes (mLN), liver, lung, gut, and skin, given significant GVHD symptoms developed in target organs in B6 to BALB/c model within 2 weeks after BMT. Consistent with the features of allogeneic T-cell response, body weight loss and donor T-cell expansion were mainly observed in allogeneic recipients (**Figure S1**). The input tissue gDNA for lymphoid and non-lymphoid organs were 960 and 3200 ng/replicate sample, respectively, according to the manufacturer’s protocol.

**Figure 1:**
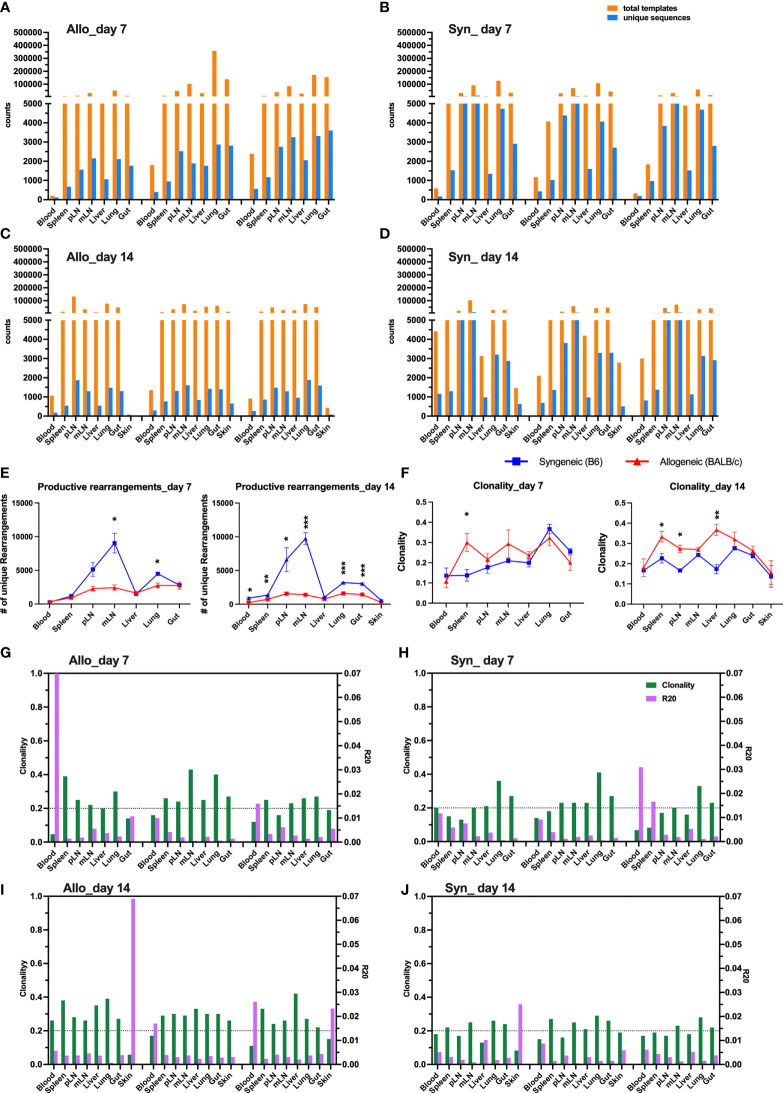
Clonal expansion of T cells in allogeneic recipient after BMT. Rag1^-/-^ BALB/c or Rag1^-/-^ B6 mice were conditioned with total body irradiation (TBI) administered 550 cGy (BALB/c) or 750 cGy (B6) using an X-RAD 320 X-ray Irradiator. Purified T cells (1 × 10^6^) from B6 mice together with bone marrow cells (5 × 10^6^) isolated form Rag1^-/-^ B6 were i.v. injected into these recipient mice within 24 h after TBI. On day 7 and day 14, gDNA were acquired from relevant tissues from individual recipient mice for TCRβ sequencing. Unique sequence was defined by CDR3 AA + V gene + J gene. The total counts of productive templates and number of unique productive sequences in each organ of individual mouse were computed and showed in bar group **(A–D)**. Data from three replicate mice were pooled for showing number of unique sequences across tissue sites **(E)**. Repertoire diversity was analyzed by calculating clonality (entropy divided by maximum possible entropy) and R20 (the minimum clone fraction required to capture 20% of overall clone frequency) in each tissue of individual mice **(G–J)**. The clonality data pooled from three replicate mice were shown **(F)**. Clonality ranges from 0 to 1 where higher scores indicate greater clonal expansion and lower repertoire diversity. R20 < 0.2 indicates non-uniform clone frequency, with extremely low values representing presence of a small number of highly dominant high-frequency clones. *p < 0.05, **p < 0.01, ***p < 0.001.

We first showed the total template counts in parallel with unique TCRβ sequences, defined by a combination of CDR3 amino acids (AA) and V and J families in three replicate mice. As an example, the TCRβ motifs of top 10 clones across all the samples tested are shown in **Figure S2**. Similar total template counts were observed comparing syngeneic with allogeneic recipients on day 7 and day 14 after BMT (**Figures 1A–D**), but the counts of unique sequences in allogenic recipients were much lower than those in syngeneic recipients, suggesting that donor T cells had more clonal expansion in allogeneic compared to syngeneic recipients in general. The mLN and lungs had significantly fewer unique sequences in allogeneic mice on day 7 after BMT, and the difference was extended to all organs except liver on day 14 (**Figure 1E**). We further studied diversity of TCRβ sequences across different recipients’ organs by analyzing clonality and R20, the minimum clone fraction required to capture 20% of overall clone frequency (**Figures 1G–J**) (13). Higher clonality and lower R20 correlate with lower diversity and more clonal expansion. TCRβ sequences in allogeneic mice had higher clonality in spleen on day 7 and the difference was further expanded to pLN and liver on day 14 (**Figure 1F**). Consistently, the accumulated frequency of the top 10–30 clones in total productive templates was increased in spleen on day 7 and also in pLN, mLN, and liver of allogeneic recipients on day 14 after BMT (**Figure S3**). Thus, we found that TCR diversity was overall narrowed accompanied with fewer unique clones but more dominant clones in recipient lymphoid and GVHD target organs, indicating clonal expansion of donor T cells after allogeneic BMT.

### Highly Overlapped TCR Clones Were Found Across Organs After Allogeneic BMT

**Figure 2:**
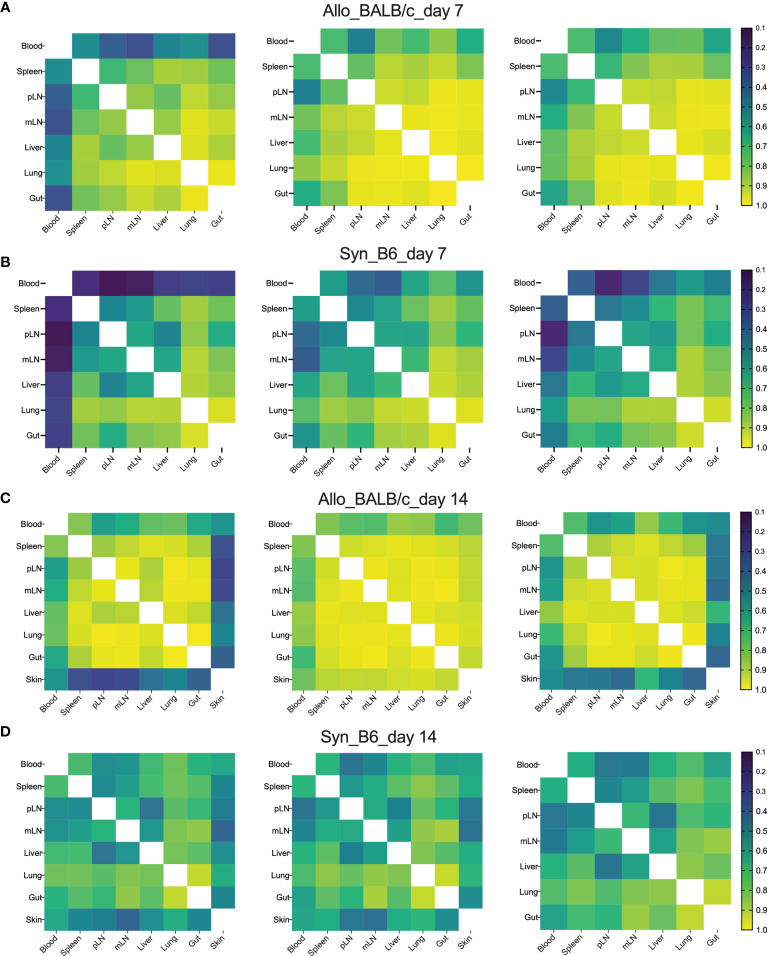
High clone sharing by different tissue sites in allogeneic recipients. Sample overlap was computed by averaging across the two ratios of shared reads over total reads for each sample. Heatmap shows the overlap metric across different tissue sites in an individual recipient on day 7 **(A, B)** and day 14 **(C, D)** after BMT.

To visualize the overall degree of TCRβ sequence sharing in various organs, we analyzed overlap metric by averaging across the two ratios of shared reads over total reads for each sample. Overall, among GVHD target organs, including liver, lungs, and gut, TCR repertoire showed higher degree of overlap than among lymphoid organs including spleen, pLN, and mLN in both syngeneic and allogeneic recipients on day 7 post BMT (**Figures 2A, B**). We observed increased overlap of TCRβ sequences across organs in the allogeneic vs. syngeneic recipients on both day 7 and 14 after BMT (**Figures 2A–D**). In addition, skin and blood TCR sequences had low overlap with other tissues analyzed in the allogeneic recipients, possibly related to few template counts in them. Comparing day 7 vs. 14 data, we found that clone overlap of post-transplant donor T cells from different tissue sites were overall decreased in syngeneic recipients but increased in allogeneic recipients at day 14. These results suggest that alloantigen stimulation selectively activates certain T-cell clones and may continuously enrich them after BMT.

### The Top Clones in Peripheral Blood Were Also Dominant Clones Across GVHD Target Organs in Allogeneic Recipients

**Figure 3:**
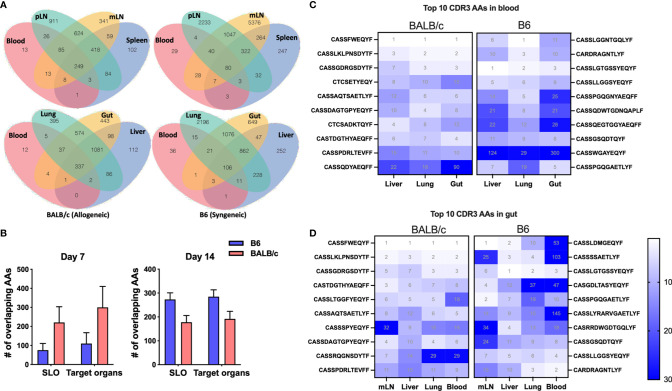
The donor T-cell clone sharing between recipient peripheral blood and GVHD target organs after BMT. Venn diagram shows the overlap between blood and secondary lymphoid organs (SLO) and GVHD target organs for the same CDR3 AAs in a representative B6 recipient (syngeneic) and allogeneic recipient (allogeneic) on day 7 after BMT **(A)**. The pooled data from replicate mice show the number of overlapped CDR3 AAs between blood and SLO or GVHD target organs on day 7 and day 14 after BMT **(B)**. Heatmap shows clone ranks of the blood-derived top 10 CDR3 AAs (from top to bottom: top 1^st^ to 10^th^) in liver, lungs, and gut in a representative recipient on day 7 after BMT **(C)**. Heatmap shows clone ranks of the gut-derived top 10 CDR3 AAs in liver, lungs, and blood in a representative recipient on day 7 after BMT **(D)**.

Since T cells in peripheral blood has the access to other tissues, we next asked whether blood samples can be used to predict major TCR clones in other organs. We first analyzed the counts of overlapped TCR clones in peripheral blood with secondary lymphoid organs (SLO) or with GVHD target organs in the same recipients, which was demonstrated using a Venn diagram to show a representative recipient and a bar graph to show the combined data from three replicate mice (**Figures 3A, B**). There were about 100 shared TCR clones in blood samples with all three SLO or GVHD target organs in syngeneic recipients on day 7 after BMT (**Figures 3A, B**). About 200–300 TCR clones in blood were found also presenting in SLO or GVHD target organs of allogeneic recipients on day 7 after BMT (**Figures 3A, B**). On day 14, absolute counts of these shared clones were increased to ~300 in syngeneic recipients while slightly reduced to ~200 in allogeneic recipients (**Figure 3B**). We found out the top 10 clones in blood samples and further analyzed the rank of these clones in individual GVHD target organs (**Figure 3C**). The top 1 and top 2 clones in blood samples remained the top 1–3 clones in the liver, lungs, and gut in allogeneic recipients. However, the top 1–2 clones in blood samples of syngeneic recipients already fell out of the top 10 clones in liver and gut in these mice. Because damage in recipient gut is critically involved in pathogenesis and development of GVHD, we also analyzed the top 10 clones in gut and studied the prevalence of these clones in mLN, blood, and other GVHD target organs. The top 1 and top 2 clones in gut remained the top 3 clones in all these organs evaluated in allogeneic recipients. However, the top 2 clones in gut fell out of the top 25 or even the top 100 clones in mLN and blood in syngeneic recipients, respectively (**Figure 3D**). Overall, these data suggest that the top clones of blood samples can be used to predict the top clones in other GVHD target organs in allogeneic recipients.

### The Dominant Clones in Allogeneic T Cells Were Not Detectable in Pre-Transplant Donor T Cells

**Figure 4:**
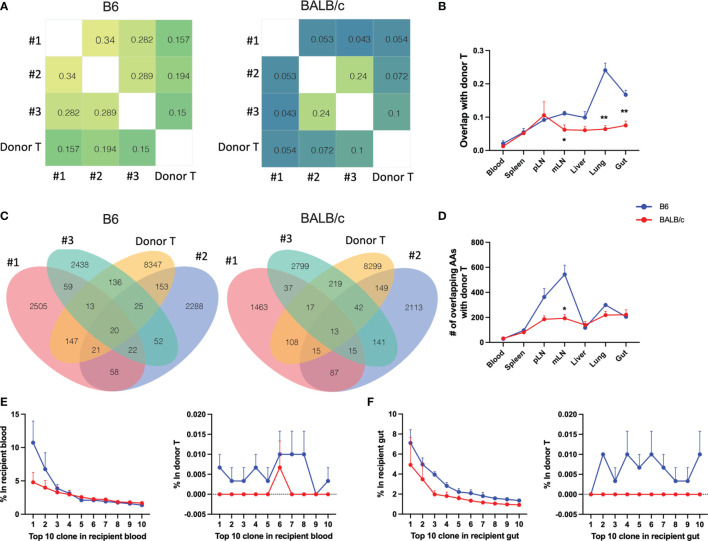
Trace back of post-transplant from pre-transplant donor T cells. Heatmap shows the overlap metric between pre-transplant donor T cells and gut T cells in an individual recipient on day 7 after BMT **(A)**. Overlap metric with pre-transplant donor T cells were further analyzed in different tissues **(B)**. Number of overlapped CDR3 AAs between pre-transplant donor T cells and gut T cells **(C)** and T cells in indicated tissues **(D)** is shown. The frequency of top 1–10 CDR3 AAs from recipient blood (**E**, left) and the frequency of these same CDR3 AAs in pre-transplant donor T cells (**E**, right). The frequency of top 1–10 CDR3 AAs from recipient gut (**F**, left) and the frequency of these same CDR3 AAs in pre-transplant donor T cells (**F**, right). *p < 0.05, **p < 0.01.

We next asked whether the T-cell clones in the recipients can be traced back in donor T cells. We analyzed the degree of TCR clone-sharing between donor T cells isolated from recipient tissues and the T cells from original grafts prior to transplant (referred to as pre-transplant donor T cells hereto after). In syngeneic recipients, T cells in blood and spleen had the lowest while lungs and gut had the highest degree of overlap with pre-transplant donor T cells. Comparing syngeneic vs. allogeneic recipients, pre-transplant donor T cells showed significantly reduced degree of repertoire overlapping with those from mLN, gut, and lungs of allogeneic recipients (**Figures 4A, B**). In the syngeneic recipients, blood and spleen had the least while pLN and mLN had the most counts of overlapping clones with pre-transplant donor T cells (**Figures 4C, D**). The absolute counts of overlapped TCR clones between T cells in recipient tissues and pre-transplant donor T cells showed a significant reduction in mLN of allogeneic vs. syngeneic recipients (**Figures 4C, D**). Next, we analyzed the prevalence of the top 10 clones in T cells from recipient blood and gut. The frequencies of individual top clones were in the range of 15%–2.5% in blood and gut, while these specific clones were at low frequency (0%–0.015%) in pre-transplant donor T cells. Interestingly, the top 10 clones in blood and gut of allogenic recipients were not detectable in pre-transplant donor T cells, which was in contrast with those from syngeneic recipients (**Figures 4E, F**). These data indicate that the top clones of donor T cells in allogeneic recipients were clonal expansion of rare sequences during allogeneic response, and they could be alloreactive T-cell clones that cause GVHD.

### Fewer TCR Clones Were Shared Among Individual Allogeneic Recipients With Only a Single TCR Clone Found Within the Top 500 Clones

**Figure 5:**
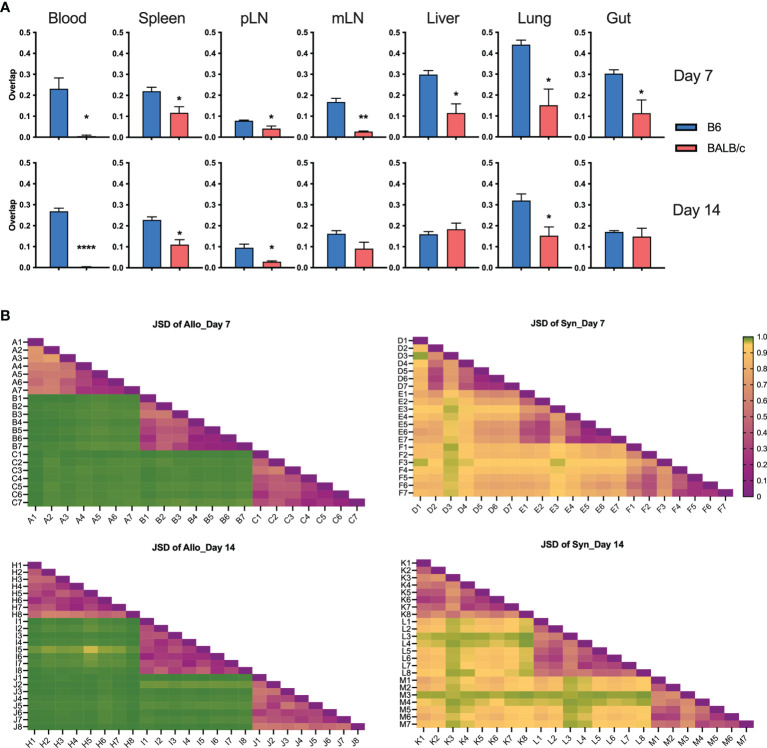
T-cell clone sharing between recipients after BMT. Bar graph shows overlap metric of T-cell clones among replicate mice **(A)**. To measure repertoire similarity, which accounts for the frequencies of shared clones, Jensen-Shannon Divergence (JSD) was computed by summing entropy(x) + entropy(y) divided by entropy of the summed vector x + y. x: Vector of clone counts or normalized frequencies for a given sample; y: Vector of clone counts or normalized frequencies for a given sample. Scales are from 0 to 1, where 0 indicates identical repertoires with identical clone frequencies and 1 indicates no shared clones. JSD data were pooled in heatmaps **(B)**. 1—blood, 2—spleen, 3—pLN, 4—mLN, 5—liver, 6—lung, 7—gut, 8—skin; capital letter represents mouse ID. *p < 0.05, **p < 0.01, ****p < 0.0001.

To understand the similarity of T-cell repertoires among recipients, we first analyzed the overlapping degree across replicate recipients in individual tissues. GVHD target organs, including lungs, liver, and gut, had the highest, while pLN and mLN had the lowest degree of TCR clone overlapping among recipients (**Figure 5A**). Compared to those in syngeneic recipients, T-cell clones in individual allogeneic recipients had significantly reduced overlap between others in most organs tested on day 7 and 14 after BMT (**Figure 5A**). We further measured TCR repertoire similarity by computing the Jensen-Shannon Divergence (JSD), which accounts for the frequencies of shared clones analyzed; 1 means totally different (low similarity); 0 means identical (high similarity) (13). By putting three replicate mice in the same heatmap, we observed a clear separation of TCR repertoire similarity in three replicate mice and little overlapping among the allogeneic recipients (**Figure 5B**).

**Figure 6:**
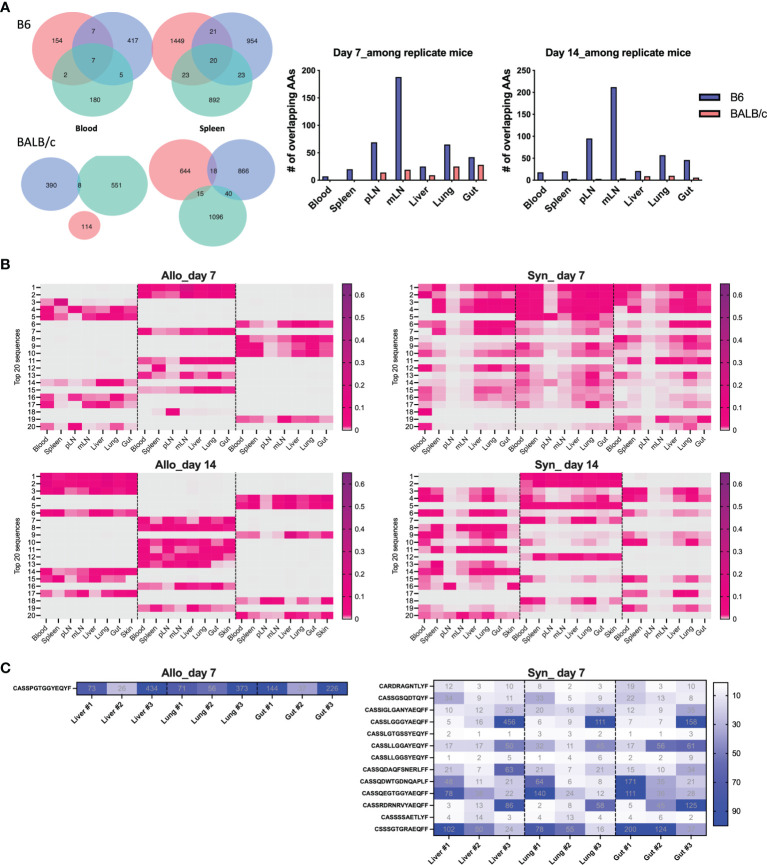
Define mouse-specific top T-cell clones after allogeneic BMT. Venn diagram shows the number of overlapped CDR3 AAs among replicate recipients on day 7 post BMT **(A)**. Top 20 sequences ranking by combined frequency in all tissues in a designated recipient group. The frequency of these clones in designated tissues from individual recipients are shown in the heatmap **(B)**. The shared clones within top 500 CDR3 AAs and among replicate recipients were listed and the clone rank of these clones are shown in liver, lung, and gut on day 7 after BMT **(C)**.

Furthermore, on day 7 post BMT, the absolute numbers of overlapped T-cell clones among three replicate recipients were much lower in the allogeneic vs. syngeneic groups across all organs, which were further reduced on day 14 after BMT (**Figure 6A**). We further studied the similarity of top 20 clones ranking by combined frequency in all tissues in a designated recipient group (**Figure 6B**). The top dominant sequences in allogeneic recipients were unique to individual recipients but shared across tissue sites. Top dominant sequences in syngeneic recipients were more commonly shared among recipient replicates and across tissue sites.

We further studied the rank of the shared clone among replicate recipients and we can only detect 1 and 17 TCRβ sequences that were shared by the replicate allogeneic and syngeneic recipients within the top 500 clones, respectively. Interestingly, this common clone shared among replicate allogeneic recipients was exactly the same in liver, lung, and gut and had the CDR3 AA sequence as CASSPGTGGYEQYF (**Figure 6C**). Among 17 common clones in syngeneic recipients, we found that 13 of them were shared across liver, lung, and gut. Overall, our data suggest that fewer T-cell clones were shared among allogeneic recipients with very low frequency among total TCR rearrangements. The dominant T-cell clones activated by alloantigen stimulation were highly recipient specific even with the same MHC disparity between donor and host after allogeneic BMT.

### V Genes Were Differentially Used by TCR Clones in Allogeneic vs. Syngeneic Recipients

**Figure 7:**
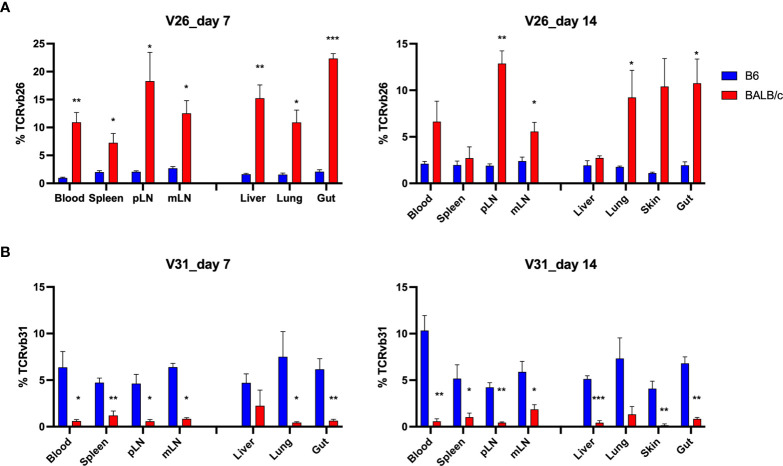
Distinct V gene usage pattern in allogeneic vs. syngeneic T cells. Frequency of V genes in total counts were analyzed in different tissues. Pooled data of V26 and V31 gene frequency are shown in the bar graph **(A, B)**. *p < 0.05, **p < 0.01, ***p < 0.001.

To understand the feature of V and J gene usage by the TCR clones, we calculated the sum frequencies of productive rearrangements that contained the specific V and D genes. Interestingly, although few TCR clones were shared among allogeneic recipients, we found that allogeneic T cells consistently used V26 more frequently than syngeneic T cells across recipient organs on days 7 and 14 after BMT (**Figure 7A**). In addition, syngeneic T cells applied V31 more frequently than allogeneic T cells (**Figure 7B**). We found that within the top 20 clones, V12, 13, 16, 19, and 29 were frequently used by donor T cells in both allogeneic and syngeneic recipients. Four clones within the top 20 clones in allogeneic T cells contained V26, while none of them contained V31. In contrast, none of top 20 clones in syngeneic T cells used V26 (data not shown). The D gene usage was comparable between these two groups (data not shown). These data suggest that the common structure of the discrepant antigens in allogeneic recipients may trigger TCR recombinant process using more V26 genes but fewer V31 genes to generate dominant clones during T-cell activation.

## Discussion

In the current study, we used murine models of BMT and extensively studied post-transplant donor T-cell repertoire in multiple lymphoid and non-lymphoid GVHD target organs. T-cell clonality, which is inversely associated with TCR diversity, was significantly increased in lymphoid organs of allogeneic compared with syngeneic recipients. T-cell clones were highly overlapped across tissue sites, especially among GVHD target organs, in the same recipients after allogeneic BMT. On the other hand, fewer common clones were shared among allogeneic recipients. Therefore, it is nearly impossible to define common pathogenetic clones that correlate to GVHD pathogenesis in different recipients. However, blood sample may be used to predict the dominant clones in GVHD target organs at the early stage after BMT. Detection of high-frequency clones that are presented in recipient blood or other tissues but not detectable in pre-transplant donor T cells may provide a novel method to predict GVHD development in clinic.

We found that an allogeneic recipient had narrowed T-cell repertoire diversity and highly overlapped TCRβ clones across lymphoid and GVHD target organs (**Figures 1**, **2**). The top clones in blood samples were also dominant clones in liver, lungs, and gut in mice with GVHD (**Figure 3C**). In an excellent study, T-cell repertoire was examined in patients with acute gastro-intestinal (GI) GVHD (12). The authors found that patients with steroid refractory GI GVHD had a more common TCRβ clonal structure between colon and upper GI tract than steroid-responsive patients. Interestingly, T-cell clones identified in the GI tract of the patients with steroid-refractory acute GI GVHD were found in their peripheral blood with disease progression. Thus, the feature of TCR repertoire diversity and clone overlap between tissue sites in a recipient seems correlated to not only the development and severity of GVHD, but also the response to steroid treatment. Therefore, monitoring the longitudinal expansion of tissue-infiltrating clones in blood could be developed as a novel biomarker of GVHD disease activity. However, it is also reported that frequent clonotypes differ among tissues in the same patient with GVHD (14). Thus, the similarity of T-cell clones from tissue to tissue needs to be further studied in GVHD patients that might be affected by anatomy location of biopsy, stage after HCT, severity of GVHD, and treatment response.

In our study, very few clones were found sharing among individual allogeneic recipients. The dominant clones were completely different from mouse to mouse and there was only one single clone found within the top 500 clones shared by replicate recipients after allogeneic BMT (**Figures 5** and **6**). By studying TCR repertoire in the GI tract of different recipients across a spectrum of matching, from syngeneic, to minor histocompatibility antigen mismatch, to major MHC mismatched, a recent study showed that TCR repertoire reconstitution follows identifiable patterns of repertoire reconstitution depending on the degree of recipient MHC match (15). They found that TCR reconstitution is highly stochastic and likely does not depend on the evaluation of the most expanded TCR clones in any individual recipient but instead depends on a complex polyclonal architecture. Similarly, the tissue-infiltrating TCRβ repertoires are highly patient specific after HCT (12, 14). Interestingly, although the dominant clones were unique to each patient, GVHD patients shared a small number of clones that were exclusively found in steroid-refractory case (16). In addition, we think the stage of disease could also impact the extent of clone sharing from recipient to recipient, which was found to be further reduced in allogeneic mice on day 14 compared to day 7 after BMT (**Figure 6A**). There was no shared clone within top 500 rearrangements among 3 replicate allogeneic recipients on day 14 (data not shown). On the other hand, we consistently found a different frequency of TCR V genes in total templates, including V26 and V31 usage in allogeneic vs. syngeneic T cells on days 7 and 14 (**Figure 7**). It needs to be further addressed whether a common structure of alloantigen from replicate mice might trigger a similar way to select V genes and form TCR structure during allogeneic T-cell activation. It was reported that there was a correlation between the presence of Vß 20 and Vß29 and steroid responsiveness in patients with GVHD (16). Thus, V gene usage and also VJ pairing in T-cell repertoire may be useful to define biomarkers that can quantitatively assess the risk of GVHD development or treatment failure.

The top 10 clones in blood and gut of allogeneic recipients were not detectable in pre-transplant donor T cells (**Figures 4E, F**), suggesting that these might be the alloreactive clones that were rarely presented in pretransplant-donor T cells, but were highly expanded in response to alloantigen stimulation that might contribute to GVHD pathogenesis. In a clinical study of 135 serial specimens from a cohort of 35 allo-HCT recipients/donors, rarefied and hyperexpanded clonotypic patterns were found to be the hallmarks of T-cell reconstitution and influenced clinical outcomes (17). By using CDR3-based specificity spectrum analysis, they found dominant expansion of pathogen- and tumor-associated clonotypes in the late post–allo-HCT phase, while autoreactive clones were more expanded in the case of GVHD occurrence. Further study is needed to confirm the alloreactivity of these clones by using mixed lymphocyte reaction (MLR) experiments and testing their reactivity to recipient cells. A previous study used MLR experiments to define donor-reactive T-cell repertoires before solid organ transplant. The authors traced these donor-reactive clones after combined kidney and BMT transplant and found the reduction of these clones in tolerant patients but not in nontolerant patients (6). Therefore, it is critical to identify the fingerprint of alloreactive T-cell clones in pre-transplant donor T cells and further study their prevalence, phenotype, and function after allogeneic BMT.

To focus our study on the repertoire of donor-derived T cells and exclude the influence of residual host T cells early after BMT, we used Rag1^-/-^ mice as recipients. Rag1^-/-^ mice have a greatly impaired production of mature T cells or B cells due to deletion of a multiprotein RAG complex involved in V(D)J recombination (18). However, other immune components are complete in these mice, including antigen-presenting cells (APC) and NK cells. The APC function of Rag1^-/-^ mice is intact or even elevated due to lack of Treg control of B7 expression on dendritic cells (19). It was reported that reconstitution of syngeneic, naïve (CD45RB^hi^ or CD62L^+^CD44^-^) CD4 T cells in these lymphopenic mice causes an oligoclonal expansion of effector CD4 T cells and colitis development due to their cross-reactivities with endogenous antigens, including self-antigens and gut microflora (20, 21). In our study, purified total T cells from B6 mice were able to respond to alloantigens, causing GVHD clinical symptom in allogeneic Rag1^-/-^ recipients, reflected by the reduced body weight of the BALB/c Rag1^-/-^ recipients. However, the syngeneic B6 Rag1^-/-^ recipients receiving the same donor T cells maintained almost 100% body weight after BMT (**Figure S1A**). The relative low number of total T cells containing a mixture of naïve/memory and CD4/CD8 T cells were transferred into recipient mice which were insufficient to cause colitis in B6 Rag1^-/-^ recipients. Although not perfect models to represent a clinical scenario, the TCR repertoire profile generated from our study can largely reflect the biology of T-cell allogeneic response and GVHD pathogenesis at TCR clone levels. Indeed, consistent with our observation, a clinical study using limited specimen also had similar results regarding highly patient-specific TCRβ repertoires and TCR clone-sharing among anatomy location in a patient after HCT.

In short, our study highlights the importance of T-cell repertoire profiling in understanding the biology of allogeneic T-cell response post BMT. A comprehensive patient-specific T-cell repertoire profiling in combination with single-cell RNA sequence may provide a deeper understanding to the correlation of T-cell repertoire with T-cell differentiation, function, and tissue infiltration during GVHD development, which may provide insightful knowledge to improve clinical diagnosis and treatment for patients after allogeneic HCT.

## Data Availability Statement

The raw sequence data for the 90 generated libraries were deposited at the Sequence Read Archive: SRR16914563, SRR16914564, SRR16914565, SRR16914566, SRR16914482, SRR16914483, SRR16914484, SRR16914485, SRR16914486, SRR16914487, SRR16914489, SRR16914490, SRR16914491, SRR16914492, SRR16914493, SRR16914494, SRR16914495, SRR16914496, SRR16914497, SRR16914498, SRR16914500, SRR16914501, SRR16914502, SRR16914503, SRR16914504, SRR16914505, SRR16914506, SRR16914507, SRR16914508, SRR16914509, SRR16914511, SRR16914512, SRR16914513, SRR16914514, SRR16914515, SRR16914516, SRR16914517, SRR16914518, SRR16914519, SRR16914520, SRR16914522, SRR16914523, SRR16914524, SRR16914525, SRR16914526, SRR16914527, SRR16914528, SRR16914529, SRR16914530, SRR16914531, SRR16914533, SRR16914534, SRR16914535, SRR16914536, SRR16914537, SRR16914538, SRR16914539, SRR16914540, SRR16914541, SRR16914542, SRR16914544, SRR16914545, SRR16914546, SRR16914547, SRR16914548, SRR16914549, SRR16914550, SRR16914551, SRR16914552, SRR16914553, SRR16914555, SRR16914556, SRR16914557, SRR16914558, SRR16914559, SRR16914560, SRR16914561, SRR16914567, SRR16914568, SRR16914569, SRR16914562, SRR16914488, SRR16914499, SRR16914510, SRR16914521, SRR16914532, SRR16914543, SRR16914554, SRR16914570, SRR16914571.

## Ethics Statement

The animal study was reviewed and approved by the Medical University of South Carolina.

## Author Contributions

YW performed research, collected, analyzed and interpreted data, and drafted and revised the manuscript. JF analyzed data and edited the manuscript. HW interpreted data and edited the manuscript. X-ZY designed the research, interpreted data, and revised the manuscript. All authors contributed to the article and approved the submitted version.

## Funding

This work was supported by NIH Grants R01s AI118305, HL137373, and HL140953, and by SC SmartState endowment funds to X-ZY.

## Conflict of Interest

The authors declare that the research was conducted in the absence of any commercial or financial relationships that could be construed as a potential conflict of interest.

## Publisher’s Note

All claims expressed in this article are solely those of the authors and do not necessarily represent those of their affiliated organizations, or those of the publisher, the editors and the reviewers. Any product that may be evaluated in this article, or claim that may be made by its manufacturer, is not guaranteed or endorsed by the publisher.

## Supplementary Material

Supplementary Figure 1 Body weight change and donor T cell engraftment after BMT. Rag1^-/-^ BALB/c or Rag1^-/-^ B6 mice were conditioned with total body irradiation (TBI) administered 550cGy (BALB/c) or 750cGy (B6) using an X-RAD 320 X-ray Irradiator. Purified T cells (1×10^6^) from CD45.1^+^ B6 mice together with bone marrow cells (5×10^6^) isolated form Rag1^-/-^ B6 were i.v. injected into these recipient mice within 24h after TBI. Recipient body weight are shown **(A)**. On day 7 **(B)** and day 14 **(D)**, recipient splenocytes were subjected for flow testing of H2K^b^ expression on gated live cells and TCRvβ and CD45.1 expression on gated H2K^b+^ live cells from 3 replicate recipients.

Supplementary Figure 2 Top 10 clones in all recipients after BMT. Rag1^-/-^ BALB/c or Rag1^-/-^ B6 mice were conditioned with total body irradiation (TBI) administered 550cGy (BALB/c) or 750cGy (B6) using an X-RAD 320 X-ray Irradiator. Purified T cells (1×10^6^) from CD45.1^+^ B6 mice together with bone marrow cells (5×10^6^) isolated form Rag1^-/-^ B6 were i.v. injected into these recipient mice within 24h after TBI. On day 7 and day 14, gDNA were acquired from relevant tissues from individual recipient mice for TCRβ sequencing. Unique sequence was defined by CDR3 amino acid + V gene + J gene. The structure of the top 10 clones, sum of template and number of samples containing the top 10 clones are shown across all the samples tested.

Supplementary Figure 3 Frequency of top clones in recipients after BMT. Rag1^-/-^ BALB/c or Rag1^-/-^ B6 mice were conditioned with total body irradiation (TBI) administered 550cGy (BALB/c) or 750cGy (B6) using an X-RAD 320 X-ray Irradiator. Purified T cells (1×10^6^) from B6 mice together with bone marrow cells (5×10^6^) isolated form Rag1^-/-^ B6 were i.v. injected into these recipient mice within 24h after TBI. On day 7 and day 14, gDNA were acquired from relevant tissues from individual recipient mice for TCRβ sequencing. The frequency of top 5, 10, 20 and 30 clones in total templates were analyzed in each recipient organs after BMT **(A, B)**.
