# Supplementary material for: Donor T-Cell Repertoire Profiling in Recipient Lymphoid and Parenchyma Organs Reveals GVHD Pathogenesis at Clonal Levels After Bone Marrow Transplantation in Mice
Source: Front Immunol. 2021 Dec 7;12:778996. doi: 10.3389/fimmu.2021.778996 (PMC8688739; doi:10.3389/fimmu.2021.778996)
Supplement: Supplementary Figure 1 — Body weight change and donor T cell engraftment after BMT. Rag1-/- BALB/c or Rag1-/- B6 mice were conditioned with total body irradiation (TBI) administered 550cGy (BALB/c) or 750cGy (B6) using an X-RAD 320 X-ray Irradiator. Purified T cells (1×10^6^) from CD45.1+ B6 mice together with bone marrow cells (5×10^6^) isolated form Rag1-/- B6 were i.v. injected into these recipient mice within 24h after TBI. Recipient body weight are shown (A). On day 7 (B) and day 14 (D), recipient splenocytes were subjected for flow testing of H2Kb expression on gated live cells and TCRvβ and CD45.1 expression on gated H2Kb+ live cells from 3 replicate recipients. [file DataSheet_1.pdf]

Figure S1

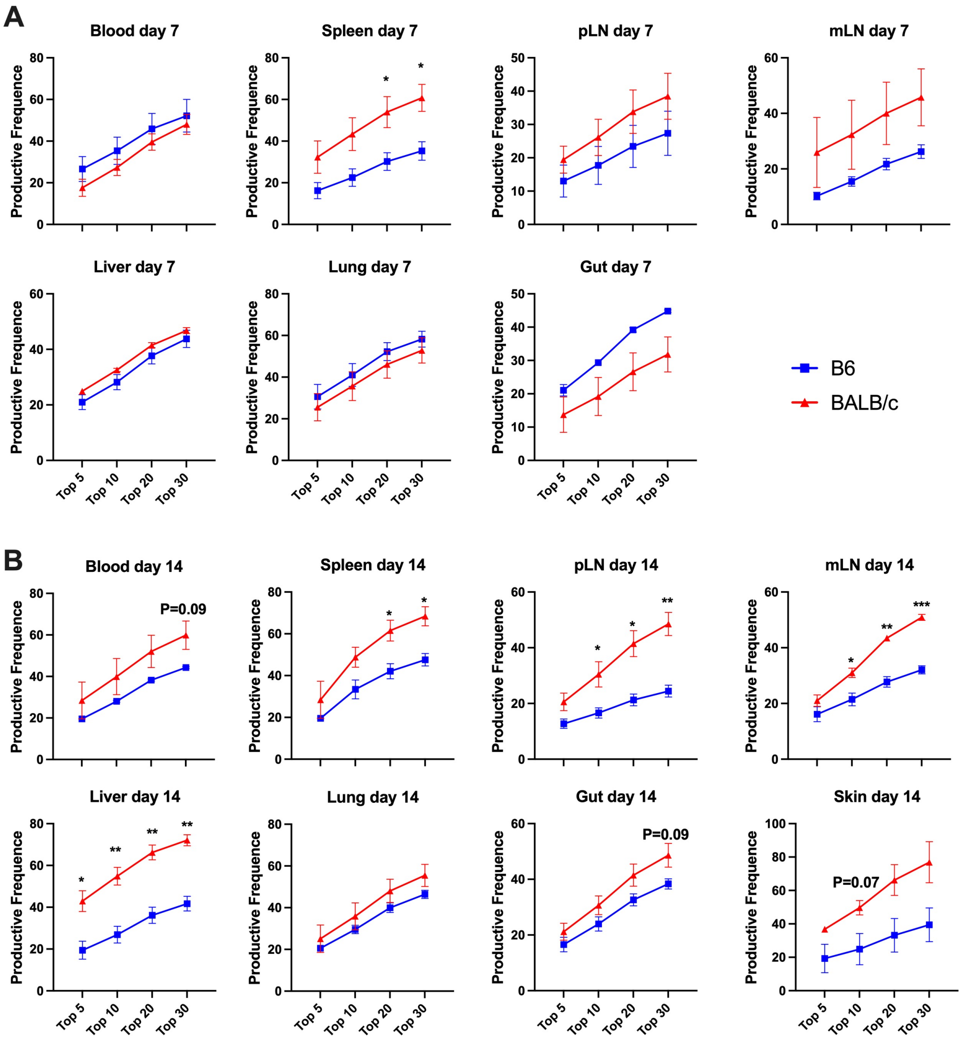

Figure S1. Body weight change and donor T cell engraftment after BMT. Rag1$^{-/-}$ BALB/c or Rag1$^{-/-}$ B6 mice were conditioned with total body irradiation (TBI) administered 550cGy (BALB/c) or 750cGy (B6) using an X-RAD 320 X-ray Irradiator. Purified T cells (1×10^6) from CD45.1$^+$ B6 mice together with bone marrow cells (5×10^6) isolated form Rag1$^{-/-}$ B6 were i.v. injected into these recipient mice within 24h after TBI. Recipient body weight are shown **(A)**. On day 7 **(B)** and day 14 **(D)**, recipient splenocytes were subjected for flow testing of H2K^b expression on gated live cells and TCRvβ and CD45.1 expression on gated H2K^{b+} live cells from 3 replicate recipients.

Figure S2

Bioidentity (top 10 in all)	Sum (Templates)	Present In
CASSFWEQYF+TCRBV29-01+TCRBJ02-07	104054	21
CASSLKLPNSDYTF+TCRBV15-01+TCRBJ01-02	62504	20
CASSLGTGSSYEQYF+TCRBV12-01+TCRBJ02-07	43005	51
CASSLLGGSYEQYF+TCRBV03-01+TCRBJ02-07	30851	49
CARDRAGNTLYF+TCRBV31-01+TCRBJ01-03	29135	46
CGARAENYAEQFF+TCRBV20-01+TCRBJ02-01	27097	17
CASSGDRGSDYTF+TCRBV13-01+TCRBJ01-02	24276	11
CASSDAGTGPYEQYF+TCRBV13-03+TCRBJ02-07	21183	13
CASSLTGGFYEQYF+TCRBV19-01+TCRBJ02-07	16509	10
CASSSGTGVTGQLYF+TCRBV29-01+TCRBJ02-02	16405	12

Figure S2. Top 10 clones in all recipients after BMT. Rag1$^{-/-}$ BALB/c or Rag1$^{-/-}$ B6 mice were conditioned with total body irradiation (TBI) administered 550cGy (BALB/c) or 750cGy (B6) using an X-RAD 320 X-ray Irradiator. Purified T cells (1×10^6) from CD45.1$^+$ B6 mice together with bone marrow cells (5×10^6) isolated form Rag1$^{-/-}$ B6 were i.v. injected into these recipient mice within 24h after TBI. On day 7 and day 14, gDNA were acquired from relevant tissues from individual recipient mice for TCRβ sequencing. Unique sequence was defined by CDR3 amino acid + V gene + J gene. The structure of the top 10 clones, sum of template and number of samples containing the top 10 clones are shown across all the samples tested.

Figure S3

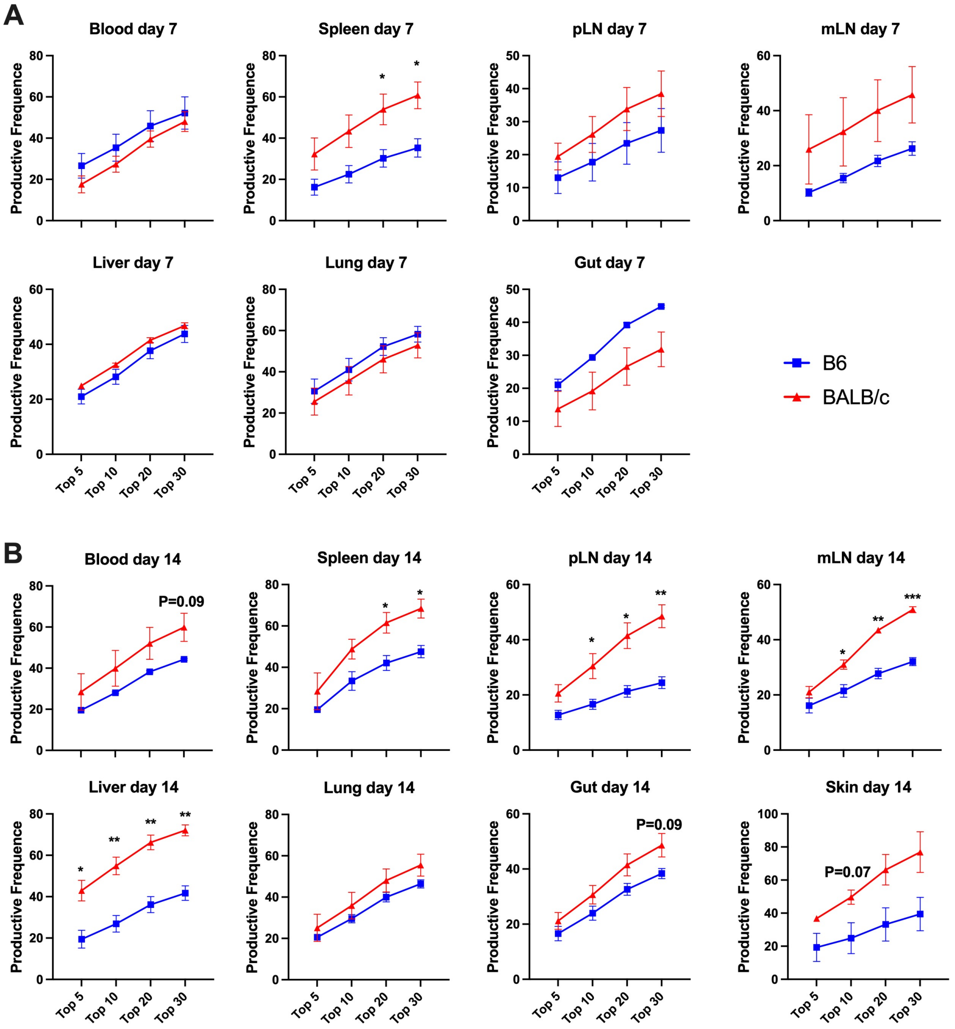

Figure S3. Frequency of top clones in recipients after BMT. Rag1$^{-/-}$ BALB/c or Rag1$^{-/-}$ B6 mice were conditioned with total body irradiation (TBI) administered 550cGy (BALB/c) or 750cGy (B6) using an X-RAD 320 X-ray Irradiator. Purified T cells (1×10^6) from B6 mice together with bone marrow cells (5×10^6) isolated form Rag1$^{-/-}$ B6 were i.v. injected into these recipient mice within 24h after TBI. On day 7 and day 14, gDNA were acquired from relevant tissues from individual recipient mice for TCRβ sequencing. The frequency of top 5, 10, 20 and 30 clones in total templates were analyzed in each recipient organs after BMT (A-B).
